# Optimal dosing of antibiotics in critically ill patients by using continuous/extended infusions: a systematic review and meta-analysis

**DOI:** 10.1186/cc13134

**Published:** 2013-11-29

**Authors:** Clarence Chant, Ann Leung, Jan O Friedrich

**Affiliations:** 1 Pharmacy Department, St. Michael’s Hospital, Toronto, Canada; 2 Li Ka Shing Knowledge Institute, St. Michael’s Hospital, Toronto, Canada; 3 Interdepartmental Division of Critical Care, University of Toronto, Toronto, Canada; 4 Critical Care and Medicine Departments, St. Michael’s Hospital and University of Toronto, 30 Bond Street, Bond Wing, Room 4-015, Toronto, Ontario M5B 1W8, Canada

## Abstract

**Introduction:**

The aim of this study was to determine whether using pharmacodynamic-based dosing of antimicrobials, such as extended/continuous infusions, in critically ill patients is associated with improved outcomes as compared with traditional dosing methods.

**Methods:**

We searched Medline, HealthStar, EMBASE, Cochrane Clinical Trial Registry, and CINAHL from inception to September 2013 without language restrictions for studies comparing the use of extended/continuous infusions with traditional dosing. Two authors independently selected studies, extracted data on methodology and outcomes, and performed quality assessment. Meta-analyses were performed by using random-effects models.

**Results:**

Of 1,319 citations, 13 randomized controlled trials (RCTs) (*n* = 782 patients) and 13 cohort studies (*n* = 2,117 patients) met the inclusion criteria. Compared with traditional non-pharmacodynamic-based dosing, RCTs of continuous/extended infusions significantly reduced clinical failure rates (relative risk (RR) 0.68; 95% confidence interval (CI) 0.49 to 0.94, *P* = 0.02) and intensive care unit length of stay (mean difference, −1.5; 95% CI, −2.8 to −0.2 days, *P* = 0.02), but not mortality (RR, 0.87; 95% CI, 0.64 to 1.19; *P* = 0.38). No significant between-trial heterogeneity was found for these analyses (*I*^*2*^ = 0). Reduced mortality rates almost achieved statistical significance when the results of all included studies (RCTs and cohort studies) were pooled (RR, 0.83; 95% CI, 0.69 to 1.00; *P* = 0.054).

**Conclusions:**

Pooled results from small RCTs suggest reduced clinical failure rates and intensive care unit length-of-stay when using continuous/extended infusions of antibiotics in critically ill patients. Reduced mortality rates almost achieved statistical significance when the results of RCTs were combined with cohort studies. These results support the conduct of adequately powered RCTs to define better the utility of continuous/extended infusions in the era of antibiotic resistance.

## Introduction

Optimal use of antimicrobials is crucial in the critical care setting, especially in an era of increasing antibiotic resistance and lack of new antimicrobial development [1]. Interest is growing in alternative antimicrobial dosing strategies that are better aligned with the antimicrobial’s pharmacodynamic properties, and the potential of this approach to improve patient outcomes [2]. Given the highly variable and often unknown pharmacokinetics of antimicrobials in critically ill patients as compared with other hospitalized patients, alignment with the pharmacodynamics (PDs) of the antimicrobials is even more important [3]. Antimicrobial pharmacodynamics refers to the effects of a drug on microorganisms in relation to the drug’s concentration within the body (that is, pharmacokinetics, PCKs). The PD of beta-lactam antimicrobials (for example, penicillins, cephalosporins) are termed time-dependent, as their effects are best correlated with the amount of time that the serum concentrations of the antimicrobial are above the minimum inhibitory concentration (MIC) of the microorganism. Other antibiotics, such as fluoroquinolones and aminoglycosides, have PD properties termed concentration-dependent killing, given that their effects correlate best with peak concentration/MIC ratio and/or area under the concentration-time curve/MIC ratio [3]. To maximize microorganism eradication, several dosing methods that exploit the antimicrobial PD properties have been investigated. These include administration of time-dependent antimicrobials via extended (for example, over a period of 3 to 4 hours) or continuous infusion, as compared with traditional intermittent infusions (for example, over a period of 30 minutes), or altering doses based on both patient-specific pharmacokinetic parameters and the MIC of the target organism (also known as dual individualization) [3,4].

Unless clinical benefits are compelling, widespread clinical application of pharmacodynamics-based dosing (PDD) is unlikely, given the multitude of barriers to their implementation. These barriers include (a) identification of the types of patients that would benefit the most, with the critically ill patient population being the most obvious choice, given their heightened risk of infectious-related morbidity and mortality and increasing resistance; (b) requirement of significant practice changes in microbiology, such as routine MIC determination by using more accurate nonautomated techniques; (c) better-defined pharmacokinetics of antimicrobials in patients in the intensive care unit (ICU) with varying degrees of renal and hepatic dysfunction, as well as the extent of medication removal by a variety of renal-replacement therapies; and (d) methods to manage the need of a dedicated intravenous line for administration via continuous/extended infusions. To justify such changes, results of rigorously conducted and adequately powered RCTs in a population most likely to benefit (for instance, ICU patients) are needed, the design of which should be informed by comprehensive systematic review of current evidence. Previous systematic reviews that included both critically ill and non-critically ill patient populations have provided inconsistent results [5-7].

Therefore, to better define the current state of knowledge on this important topic and to update previously reported systematic reviews, we conducted a systematic review and meta-analysis comparing PD antimicrobial dosing with traditional non-PDD on clinical outcomes (mortality, clinical failure rates, and length-of-stay (LOS)) focusing on critically ill patients. We included both randomized and cohort studies but emphasized the results of the RCTs in the interpretation of the results.

## Materials and methods

### Data sources

With the assistance of a librarian, we systematically searched MEDLINE, HealthStar, EMBASE, Cochrane Clinical Trials Registry, and CINAHL electronically from inception (1948, 1967, 1974, 1966, and 1981, respectively) to September 24, 2013, by using the following key words: critical care, critical illness, intensive care unit, specific names of antibacterial agents, pharmacokinetic, pharmacodynamic, extended infusion, continuous infusion, drug administration, and dual individualization. Terms were “exploded” and combined by using Boolean operators where appropriate [see Additional file 1]. No language restrictions were applied. Reference lists of selected articles and personal files were also searched for relevant citations.

### Study selection

Inclusion criteria for this meta-analysis were as follows: (a) adult (older than 16 years) critically ill patients, (b) intervention that compared PDD to aid in the determination of antibiotic dosage (that is, extended infusions, continuous infusions, clinical pathway, and dual individualization principle) with a control group that did not use such dosing strategies by using either a randomized or nonrandomized study design; (c) reporting of any patient outcomes (for example, mortality, length of stay, clinical failure); and (d) any antibacterial whose PD associated with optimal killing is the proportion of time during dosing interval that is above the MIC of the pathogenic organism. Studies were excluded if (a) <50% of patients were admitted to an ICU defined by authors; (b) <50% adult patients; (c) only Monte Carlo simulation or mathematical modeling data were included; (d) no clinical outcomes were reported; (e) data were published only as an abstract; or (f) different antibiotics were used in the control/intervention groups. Citations were screened in duplicate from the initial results of the search strategy, while full-text review, also in duplicate, was performed to determine eligibility when either screening reviewer thought a citation potentially met inclusion criteria. Disagreements regarding inclusion were reconciled by consensus.

### Data extraction

A standardized data-abstraction form was designed before the conduct of the literature search. Two reviewers (CC, JF) independently abstracted data from included studies, including data on the publication (that is, year, author, and country), type of ICU, patient population, study design, interventions used (that is, antibiotic used, method of dosing), and outcomes (that is, mortality, ICU and hospital LOS, clinical failure rates). No data on harm (for example, superinfection, resistance rates) were extracted because very few studies reported such data. Risk of bias in RCTs (including blinding of participants, method of sequence generation and allocation concealment, intention-to-treat analysis, early trial stopping for efficacy before the planned enrollment was completed, and loss to follow up) and cohort studies (including retrospective versus prospective data collection, concurrent versus historical controls, and comparable baseline characteristics of cases and controls) were assessed, with disagreements resolved by consensus.

### Data analysis

Our primary outcome was all-cause mortality in patients whose infections were managed with PDD (intervention group) as compared with those whose infections were managed by antibiotic dosing that did not incorporate both pharmacodynamic and pharmacokinetic information (control group). Mortality was determined at ICU discharge, hospital discharge, 90, 60, 30, 28, or 14 days after study enrolment (in descending order of preference). Secondary outcomes were ICU and hospital LOS, and clinical failure as defined by individual study authors (for example, lack of clinical cure or improvement). Separate analyses were performed by using lack of clinical cure alone. Only RCTs were included in the primary analysis, and prespecified subgroup analyses were as follows: (a) by type of study (that is, RCT and cohort studies); (b) by antibiotic type (for example, beta-lactam alone, carbapenem alone, cephalosporin alone, piperacillin/tazobactam alone, or others); and (c) by intervention (that is, extended infusions and continuous infusions). All analyses were performed by using Review Manager (RevMan version 5.2; Cochrane Collaboration, Oxford, UK) and random effects models, which incorporate between-trial heterogeneity and give wider and more conservative confidence intervals (CIs) when heterogeneity is present [8].

We assessed statistical heterogeneity among trials by using *I*^*2*^, defined as the percentage of total variability across studies attributable to heterogeneity rather than chance, and used published guidelines for low (*I*^*2*^ = 25% to 49%), moderate (*I*^*2*^ = 50% to 74%), and high (*I*^*2*^ ≥ 75%) heterogeneity [9]. Relative risks (RRs) were used to pool binary mortality and clinical failure data, and weighted mean differences (MDs) to pool continuous LOS data. Ranges [10] and interquartile ranges [11] were converted to standard deviations by using previously published methods where necessary. Differences between pooled RRs were evaluated by using z tests. We considered (two-sided) *P* ≤ 0.05 as significant and reported individual trial and summary results with 95% confidence intervals. To assess for publication bias, we visually examined a funnel plot comparing effect measure for the primary outcome of mortality with study precision for evidence of asymmetry.

## Results

### Study selection

**Figure 1:**
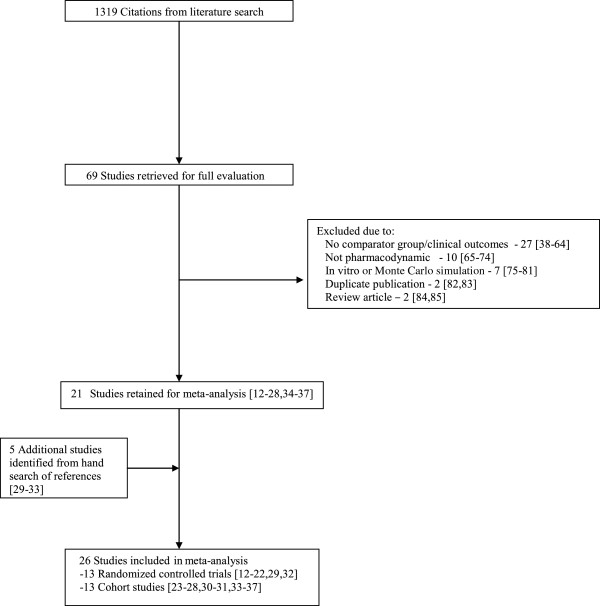
Flow chart of study selection.

In total, 26 studies were included in this meta-analysis [12-37]. The initial search strategy resulted in 1,319 citations, of which 69 were retrieved for full review and 21 met all inclusion criteria and no exclusion criteria [12-28,34-37]. Review of reference lists of the selected studies, other systematic reviews [5-7], and personal files resulted in five additional studies being included [29-33] (Figure 1). The majority of studies were excluded during initial screening because they were Monte Carlo simulation studies that did not involve patients, or were studies that did not involve PDD. The 48 studies were excluded after full review for the following reasons: lack of control group or clinical outcomes [38-64], not discussing pharmacodynamic-based dosing [65-74], Monte Carlo simulations or mathematical modeling [75-81], duplicate publications [82,83], and review articles [84,85].

### Description of included studies

The characteristics of the studies included in the meta-analysis are described in Table 1. The included studies are international (Europe, 10, USA, 11, Asia, 3, Australia, 2) with a variety of ICU patients (for example, medical, surgical, trauma, mixed) diagnosed mainly with pneumonia (*n* = 12). Most studies involved a single antibiotic (*n* = 22), typically with beta-lactam (*n* = 13) or carbapenem class (*n* = 6) or both (*n* = 4). Most used either continuous (*n* = 16) or extended (*n* = 8) infusion interventions, whereas one was a clinical pathway designed by using local antibiogram and MIC information and another by using the dual-individualization principle. Thirteen studies were RCTs, and 13 were cohort studies, of which four were prospective, and nine, retrospective. All but two of the RCTs and all but two of the non-RCTs were single center. Sample size ranged from 16 to 240 patients for the RCTs and 32 to 503 for the cohort studies.

**Table 1:** Characteristics of selected studies for meta-analysis

**Author (year) Country**	**Type of ICU**^**a**^	**Study design**	**Infection**	**Illness acuity**		**Study antibiotic**	**Control group**	**Intervention group**
				**APACHE II**	**SAPS II**			
**Randomized controlled trials**								
Georges (1999) France [12]	NR	RCT	Pneumonia or bacteremia with gram-negative bacilli		47	Cefepime	2 g q12h	4 g/d as CI
Hanes (2000) USA [13]	T	RCT	Nosocomial pneumonia	12		Ceftazidime	2 g q8h (0.5-h infusion)	LD, 2 g (0.5-h infusion), then 60 mg/kg/day as CI
Nicolau (2001) USA [14]	MS, N	RCT	VAP	15		Ceftazidime	2 g q8h (0.5-h infusion)	No LD 3 g over 24 h as CI
Wysocki (2001) France [15]	MS	RCT	Any methicillin-resistant staphylococcal infections		^b^	Vancomycin	15 mg/kg q12h (1-h infusion)	LD, 15 mg/kg over 1 h, then 30 mg/kg as CI
Bujik (2002) Netherlands [16]	S	RCT (partial)	Severe intraabdominal infection	15		Ceftazidime	1.5 g tid (20-min infusion)	LD, 1 g over 20 min, then 4.5 g/d as CI
Georges (2005) France [17]	M, T	RCT	Nosocomial pneumonia or bacteremia		45	Cefepime	2 g q12h (0.5-h infusion)	No LD; 4 g CI
Rafati (2006) Iran [18]	General	RCT	Sepsis from any source	15		Piperacillin alone	3 g q6h (0.5-h infusion)	LD, 2 g over 0.5 h, then 8 g/24 h as CI
Roberts (2007) Australia [19]	General	RCT	Sepsis from any source	18		Ceftriaxone	LD = 500 mg, then 2 g q24h	LD, 500 mg, then 2 g/24 h as CI
Sakka (2007) Germany [20]	NR	RCT	Nosocomial pneumonia	27	44	Imipenem	1 g q8h (40-min infusion)	LD, 1 g over 40 min, then 2 g/24 hr as CI for 3 days, then 1 g q8h over 40 min
Adembri (2008) Italy [21]	M, T	RCT	Sepsis; glycopeptide resistant or failure		45	Linezolid	600 mg q12h (0.5-h infusion)	LD, 300 mg, Day 1: 900 mg CI, Day 2 onward: 1,200 mg CI
Wang (2009) China [32]	NR	RCT	Acinetobacter pneumonia	19		Meropenem	1 g q8h (1-h infusion)	500 mg q6h as 3-h EI
Chytra (2012) Czech [22]	M	RCT	Severe infection from any source	22		Meropenem	2 g q8h (0.5-h infusion)	LD, 2 g over 0.5 h, then 4 g/d as CI
Dulhunty (2012) Australia [29]	NR	RCT	Severe sepsis	22		Ticarcillin/clavulanate, piperacillin/tazobactam, or meropenem	Dose determined by MD	Dose determined by MD
							All as intermittent infusion	All as CI
**Cohort studies**								
Schentag (1984) USA [23]	NR	Cohort	Gram-negative nosocomial pneumonia	NR		Cefmenoxime	Fixed dose 1–2 g q6-8 h	Integration of patient-specific PCK with bacteria-specific killing kinetics (doses ranged from 0.5 g q8h to 2 g q4h)
Lorente (2006) Spain [24]	MS	Cohort	VAP with gram-negative bacilli	15		Meropenem	1 g q6h (0.5-h infusion)	LD, 1 g over 0.5 h, then 1 g q6h as CI
Itabashi (2007) Japan [33]	NR	Cohort	Gram-negative pneumonia	NR		Meropenem	500 mg q12h (0.5- to 1-h infusion)	500 mg q12 as 4-h EI
Lodise (2007) USA [25]	NR	Cohort	Pseudomonal infections of any source	16		Piperacilin/tazobactam	3.375 g q4 or 6 h	3.375 g q8h as 4-h EI
Lorente (2007) Spain [26]	MS	Cohort	VAP with gram-negative bacilli	16		Ceftazidime	2 g q12h (0.5-h infusion)	LD, 1 g over 0.5 h, then 2 g q12h as CI
Lorente (2009) Spain [31]	MS	Cohort	VAP with gram-negative bacilli	16		Piperacillin/tazobactam	4.5 g q6h (0.5-h infusion)	LD, 4.5 g over 0.5 h, then 4.5 g q6h as CI
Nicasio (2010) USA [27]	MS, N	Cohort	VAP	19		Cefepime, or meropenem	MD discretion (0.5 h-infusions)^a^	VAP pathway derived by local MICs and PD analysis using Monte Carlo simulations (3-h infusions)
Dow (2011) USA [30]	MS	Cohort	Any infection except CF	25		Piperacillin/tazobactam, or meropenem	P/T 3.375 g q6h or Meropenem 500 mg q6h (0.5-h infusions)	P/T 3.375 g q8h as 4 h EI, Meropenem 500 mg q6h as 3-h EI
Yost (2011) USA [28]	NR	Cohort	Any gram-negative infection	~14^c^		Piperacillin/tazobactam	Variable nonextended infusions of piperacillin/tazobactam, cefepime, ceftazidime, imipenem, meropenem, doripenem	3.375 g q8h as 4-h EI
Akers (2012) USA [34]	Burn	Cohort	Gram-positive bacteremia	NR		Vancomycin	1 g q8h (dose adjustment to achieve trough levels 15–20 μg/ml)	3 g as CI (dose adjustment to achieve steady-state levels 20–25 μg/ml)
Lee (2012) USA [35]	NR	Cohort	Gram-negative infections	NR^d^		Piperacillin/tazobactam	2.25-4.5 g q6-8 h (0.5-h infusion)	3.375 g q8h as 4-h EI
Arnold (2013) USA [36]	NR	Cohort	Gram-negative infections	20		Cefepime, meropenem, or piperacillin/tazobactam	Cefepime 2 g q8h, meropenem 1 g q8h, piperacillin-tazobactam 4.5 g q6h (0.5-h infusions)	Same dose/medications as 3-h infusions
Hsaiky (2013) USA [37]	NR	Cohort	Gram-negative infections	16		Doripenem	0.5 g q8h (1-h infusion)	0.5 g q8h (4-h infusion)

M, mixed; MS, medical surgical; T, trauma; C, coronary; CV, cardiovascular; N, neurosurgical; NR, not reported. ^a^Piperacillin/tazobactam used as 24 h infusions in control group and not used in the intervention group. ^b^Only mean SAPS score [86] equal to 14 provided.

^c^Only midpoint of range provided.

^d^Median SOFA [87] score of 9.

APACHE II, mean or median acute physiology and chronic health evaluation II score of enrolled patients [88]; CI, continuous infusion; EI, extended infusion; LD, loading dose; MIC, minimum inhibitory concentration; PD, pharmacodynamic; PCK, pharmacokinetic; RCT, randomized controlled trial; SAPS II, mean or median simplified acute physiology score II score of enrolled patients [89]; SOFA, sequential organ failure assessment score [87]; VAP, ventilator-associated pneumonia.

For the 13 RCTs, only one had the participants blinded to study interventions, whereas six reported allocation concealment, and four specified that analysis was by intention-to-treat. Only three of the RCTs specifically reported that losses to follow-up were <5% of randomized patients.

For the cohort studies, only four of the 13 were prospective, and six studies used concurrent control groups. Details regarding assessment of bias among individual studies are outlined in Tables 2 and 3.

**Table 2:** Quality assessment of included randomized controlled trials

**Author (year) Country**	**Number of centres**	**Number of patients**	**Blinding**	**concealed allocation**	**Intention-to-treat analysis**	**Stopped early for benefit**	**Post randomization Withdrawal**
Georges (1999) France [12]	1	18	N	NR	NR	N	NR
Hanes (2000) USA [13]	1	32	N	NR	NR	N	Y (1 from each group)
Nicolau (2001) USA [14]	1	41	N	NR	NR	N	Y (5 from CI group and 1 from control group)
Wysocki (2001) France [15]	10	160	N	Y (consecutive sealed opaque envelopes)	Y	N	Y (15 from CI and 26 from control group)
Bujik (2002) Netherlands [16]^a^	1	18	N	NR	NR	N	NR
Georges (2005) France [17]	1	50	N	NR	NR	N	NR
Rafati (2006) Iran [18]	1	40	N	NR	NR	N	NR
Roberts (2007) Australia [19]	1	57	N	Y (sequential opaque sealed envelopes)	Y	N	N
Sakka (2007) Germany [20]	1	20	N	Y (sealed envelopes)	NR	N	NR
Adembri (2008) Italy [21]	1	16	N	Y (closed envelopes)	NR	N	Y (1 died, 1 developed ARF; group(s) not specified)
Wang (2009) China [32]	1	30	N	NR	NR	N	NR
Chyta (2012) Czech [22]	1	240	N	Y (sealed opaque envelopes)	Y	N	N for mortality and LoS, but Y (14 in CI and, 12 in control group) for cure data
Dulhunty (2012) Australia [29]	5	60	Y	Y (sequentially numbered sealed envelopes)	Y	N	N

Y, Yes; N, No; NR, not reported; CI, continuous infusion; LoS, length of stay; ARF, acute renal failure.

^a^Partial randomization: first six patients allocated to continuous-infusion group; next 12 patients randomized to continuous infusion or intermittent administration groups.

**Table 3:** Quality assessment of included cohort studies

**Author (year) Country**	**Number of centers**	**Number of patients**	**Prospective/retrospective**	**Concurrent control**	**Comparable baseline**
Schentag (1984) USA [23]	1	32	Prospective	N (historical)	NR
Lorente (2006) Spain [24]	1	89	Retrospective	Y (physician discretion)	Y
Itabashi (2007) Japan [33]	1	42	Prospective	Y (physician discretion)	Y
Lodise (2007) USA [25]	1	194	Retrospective	N (historical)	Y
Lorente (2007) Spain [26]	1	121	Retrospective	Y (physician discretion)	Y
Lorente (2009) Spain [31]	1	83	Retrospective	Y (physician discretion)	Y
Nicasio (2010) USA [27]	1 (3 separate ICUs)	168	Prospective	N (historical)	Y (except fewer intervention patients with liver disease)
Dow (2011) USA [30]	1	121	Retrospective	N (historical)	Y
Yost (2011) USA [28]	14	359	Retrospective	Y (physician discretion)	N (higher use of concomitant aminoglycosides, pseudomonas infections, and rates of positive cultures from respiratory and other sources in control patients)
Akers (2012) USA [34]	1	171	Retrospective	Y (physician discretion)	Y (except control group received ~10% lower average dose)
Lee (2012) USA [35]	2	148	Retrospective	N (historical)	Y (except control group more COPD patients, more concomitant use of fluoroquinolones and aminoglycosides, and longer (~1 d) duration and higher (~13%) cumulative dose of therapy)
Arnold (2013) USA [36]	1	503	Prospective	N (historical)	Y (except control group more COPD patients, more endotracheal (versus bronchoalveolar lavage) cultures, less *Hemophilus influenzae*, and more use of meropenem)
Hsaiky (2013) USA [37]	1	86 ^a^	Retrospective	N (historical)	Y (except control group had lower proportion of patients with positive blood cultures)

^a^Data from 86 critically ill patients of 200 enrolled hospitalized patients reported separately.

Y, Yes; N, No; NR, not reported; COPD, chronic obstructive pulmonary disease.

### Morbidity and mortality

**Figure 2:**
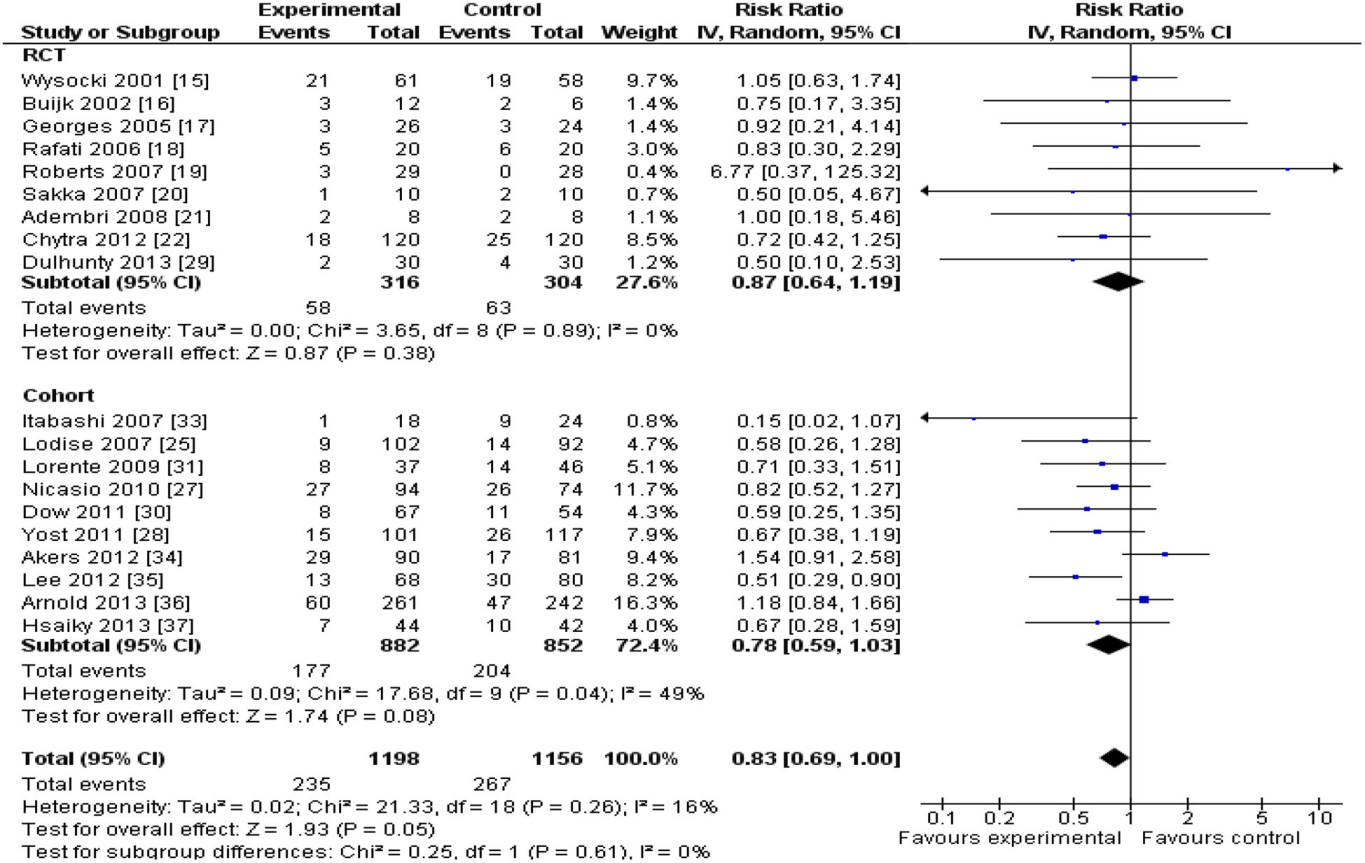
**Effects of pharmacodynamic-based antibiotic dosing on ICU**[15-17,22,29]**, hospital**[30,34,36,37]**, 14-day**[25]**, 30-day**[35]**, or unspecified (ICU or hospital)**[18-21,27,28,31,33]**mortality grouped by RCT versus cohort studies.** Individual study RRs with 95% CIs are shown as squares with lines, and pooled RRs with 95% CI, calculated by using random-effects models both overall and separately for each subgroup, are shown as diamonds. The interaction *P* value, calculated by using a *Z* test, testing for subgroup differences between the RCT and cohort studies, was not significant (*P* = 0.61). The pooled results for the RCTs were essentially unchanged if ICU mortality was replaced by the more-prolonged hospital mortality for the studies that also provided these data [22,29] (nine RCTs, 620 patients, RR, 0.86; 95% CI, 0.64 to 1.17; *P* = 0.34; *I*^*2*^ = 0%), or if the results of the partial RCT [16] were excluded (eight RCTs, 602 patients; RR, 0.88; 95% CI, 0.64 to 1.21; *P* = 0.42, *I*^*2*^ = 0%). Weight refers to the weighting of each individual study to the overall pooled RR. CI, confidence interval; IV, inverse variance; RCT, randomized controlled trial; RR, relative risk.

**Figure 3:**
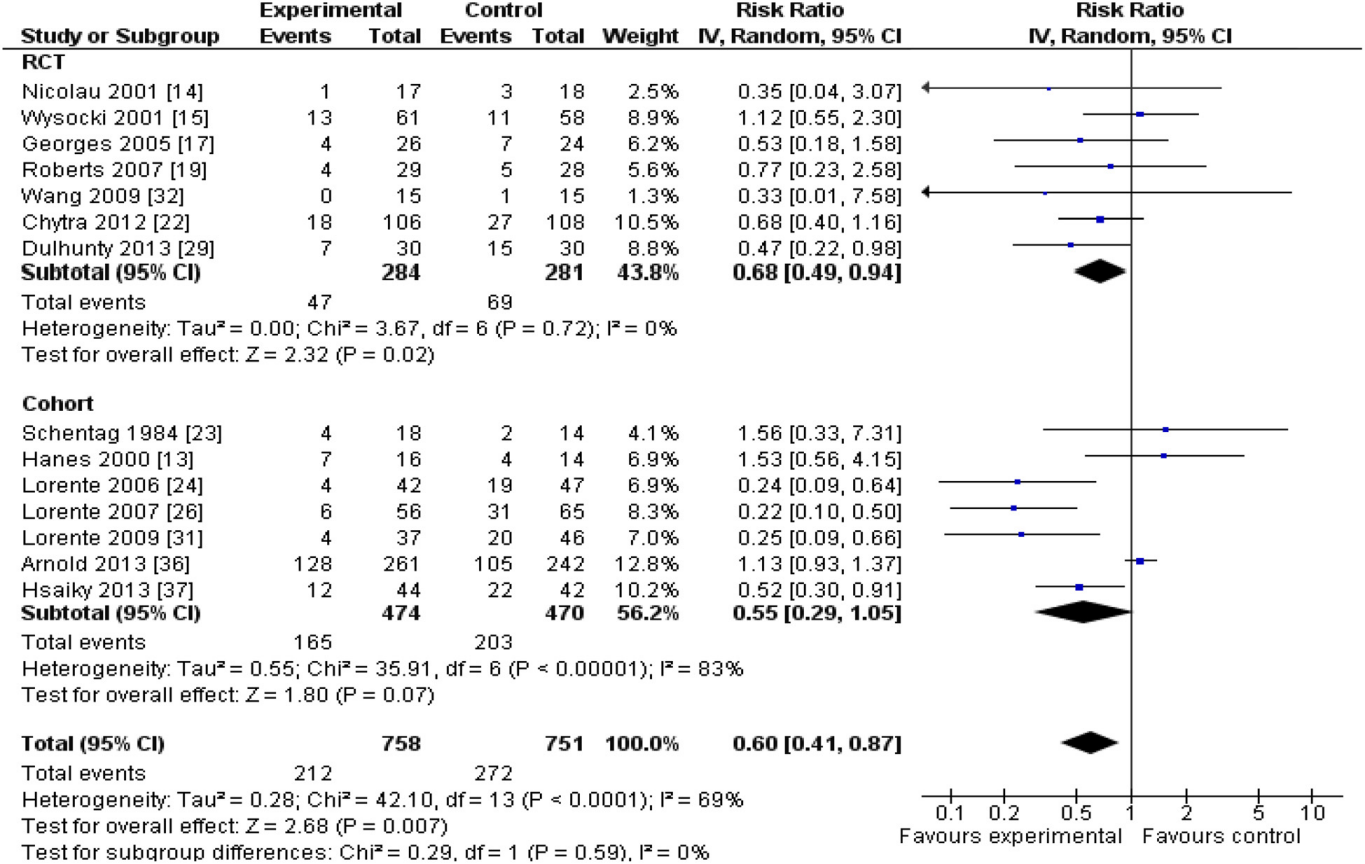
**Effects of pharmacodynamic-based antibiotic dosing on clinical failure, defined as lack of clinical cure or improvement, grouped by RCT versus cohort studies.** Individual study RR with 95% CIs are shown as squares with lines, and pooled RRs with 95% CI, calculated by using random-effects models both overall and separately for each subgroup, are shown as diamonds. Z tests were used to test for subgroup differences. If clinical failure is defined only as lack of clinical cure, results were identical for the non-RCTs and similar for the RCTs (seven RCTs, 525 patients; RR, 0.83; 95% CI, 0.70 to 0.99; *P* = 0.04; *I*^*2*^ = 11%) and overall (14 studies, 1,509 patients; RR, 0.68; 95% CI, 0.52 to 0.88; *P* = 0.004; *I*^*2*^ = 70%). Weight refers to the weighting of each individual study to the overall pooled RR. CI, confidence interval; IV, inverse variance; RCT, randomized controlled trial; RR, relative risk.

**Figure 4:**
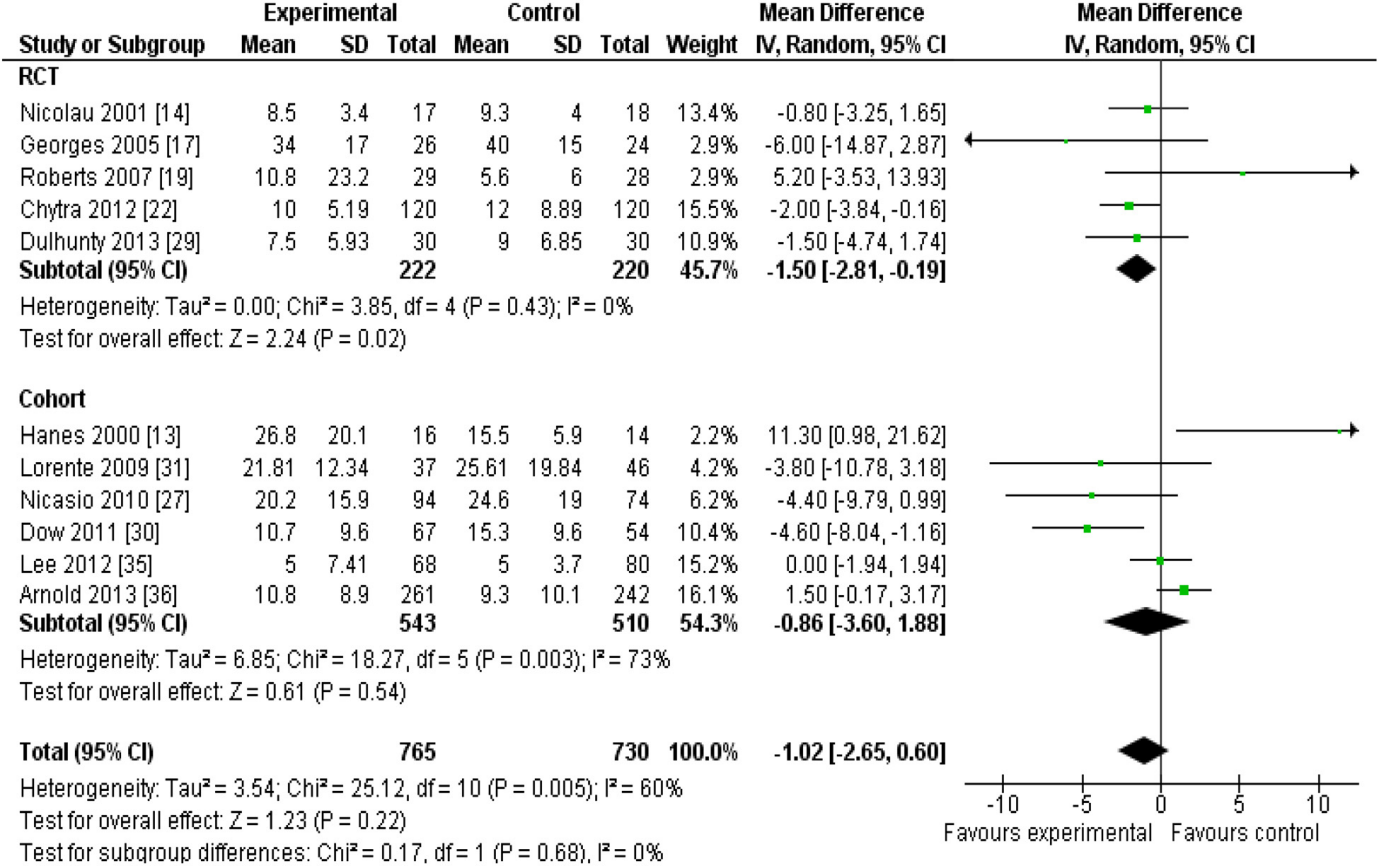
**Effects of pharmacodynamic-based antibiotic dosing on ICU length of stay, grouped by RCT versus cohort studies.** Individual study RRs with 95% CIs are shown as squares with lines, and pooled RRs with 95% CI, calculated by using random-effects models both overall and separately for each subgroup, are shown as diamonds. *Z* tests were used to test for subgroup differences. IQR [22,29,35,36] converted to standard deviations by dividing by 1.35, as previously described [11], or standard deviations calculated from reported 95% CIs, assuming equal standard deviations between groups [30]. Weight refers to the weighting of each individual study to the overall pooled RR. CI, confidence interval; IV, inverse variance; RCT, randomized controlled trial; SD, standard deviation; IQR, interquartile range.

**Figure 5:**
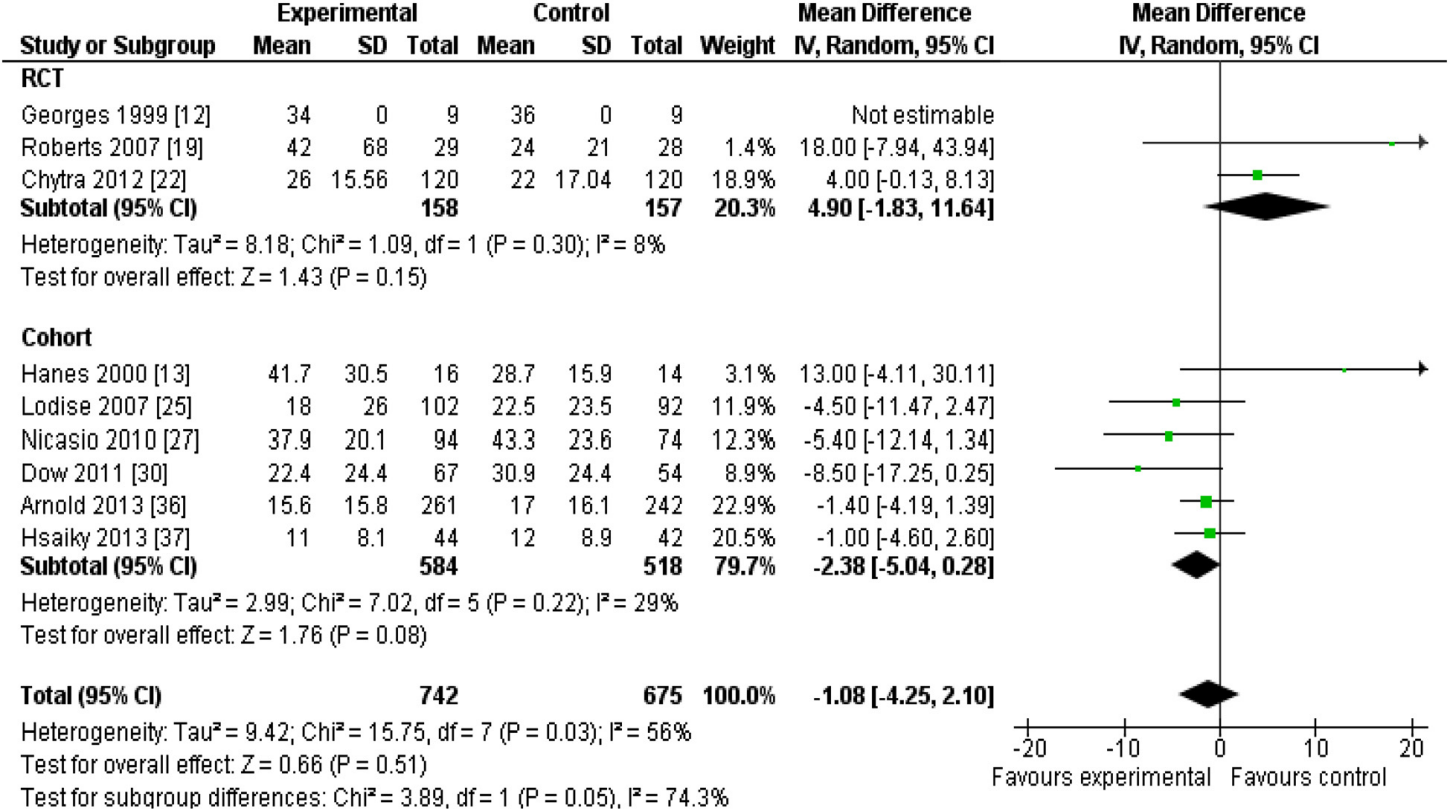
**Effects of pharmacodynamic-based antibiotic dosing on hospital length of stay, grouped by RCT versus cohort studies.** Individual study RRs with 95% CIs are shown as squares with lines, and pooled RRs with 95% CI, calculated by using random-effects models both overall and separately for each subgroup, are shown as diamonds. *Z* tests were used to test for subgroup differences. Ranges [25] or IQR [22,36,37] converted to standard deviations by using the methods of Hozo [10] or by dividing by 1.35, as previously described [11], respectively, or standard deviations calculated from reported 95% confidence intervals assuming equal standard deviations between groups [30]. Weight refers to the weighting of each individual study to the overall pooled RR. CI, confidence interval; IV, inverse variance; RCT, randomized controlled trial; SD, standard deviation; IQR, interquartile range.

The 13 RCTs [12-22,29,32] included data from 782 patients, and the 13 cohort studies [23-28,30,31,33-37], from 2,117 patients. Two studies [28,37] enrolling all hospitalized patients reported mortality data separately for patients requiring ICU admission. Reduction in mortality (nine RCTs; *n* = 620; RR, 0.87; 95% CI, 0.64 to 1.19; *P* = 0.38) almost achieved statistical significance when the results of all included studies (RCTs and cohort studies) were pooled (19 studies; n = 2,354; RR, 0.83; 95% CI, 0.69 to 1.00; *P* = 0.054) (Figure 2). Focusing the pooled analysis on only RCTs, PDD significantly reduced clinical failure rates, defined as either lack of clinical cure or improvement (seven RCTs; *n* = 565; RR, 0.68; 95% confidence interval [CI], 0.49 to 0.94; *P* = 0.02) (Figure 3), and ICU LOS (five RCTs; *n* = 442; mean difference, −1.5; 95% CI, -2.8 to −0.2 days; *P* = 0.02) (Figure 4). There was no significant between-trial heterogeneity for these analyses (*I*^*2*^ = 0). Incorporating pooled data from non-RCTs also yielded significantly reduced clinical failure rates but with increased heterogeneity (Figure 3). PDD did not result in reduced hospital lengths of stay, but few studies reported this outcome (Figure 5). Visual inspection of the funnel plot comparing the effect measure (RR) for the primary outcome of mortality for each study with its precision, expressed as the standard error of the natural logarithm of RR, SE(log(RR)) did not suggest asymmetry (see Additional file 2).

### Subgroup analysis

**Figure 6:**
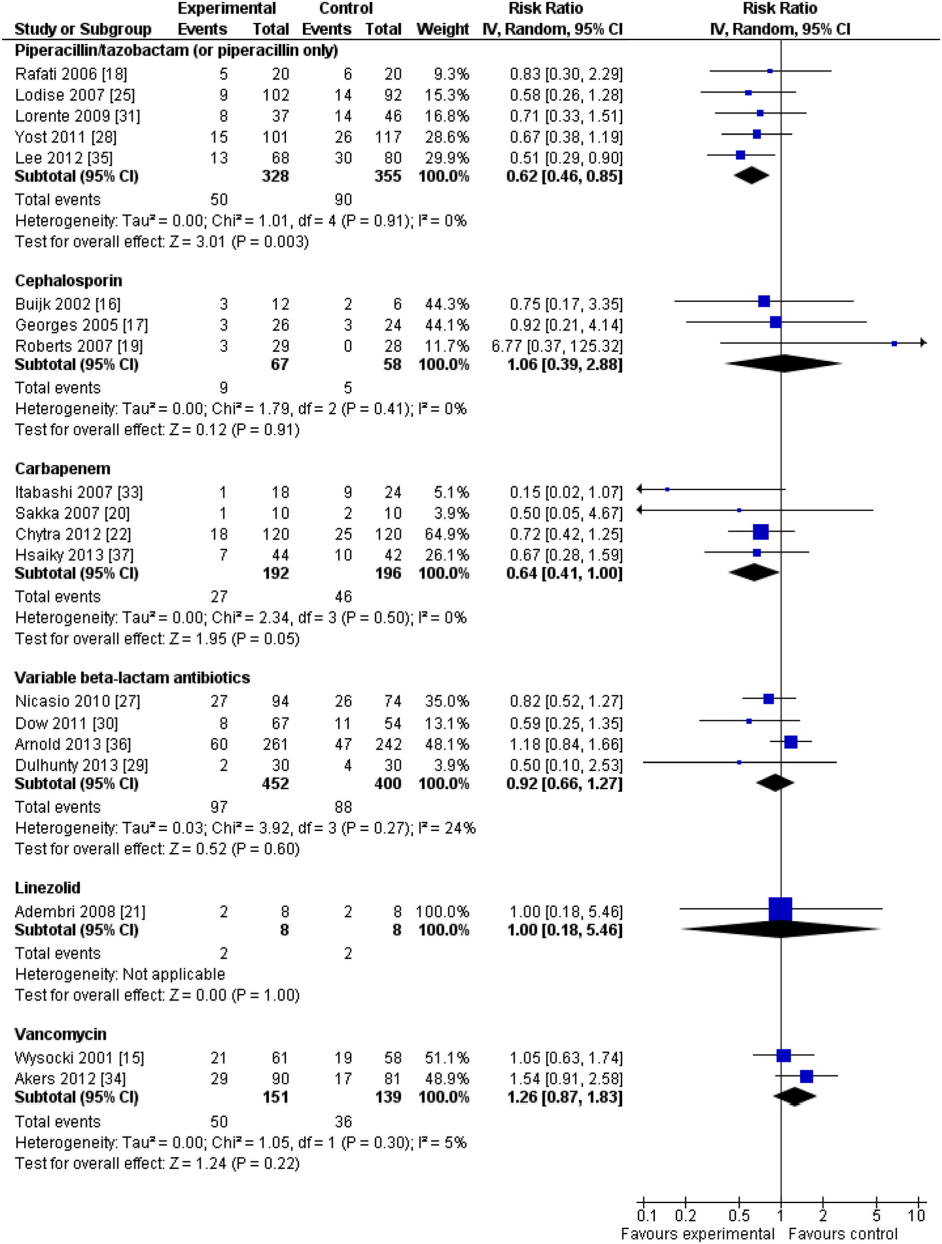
**Effects of pharmacodynamic-based antibiotic dosing on mortality separated by class of antibiotic.** Individual study RRs with 95% CIs are shown as squares with lines, and pooled RRs with 95% CI, calculated by using random-effects models separately for each class of antibiotic, are shown as diamonds. Weight refers to the weighting of each individual study to the overall pooled RR. CI, confidence interval; IV, inverse variance; RR, relative risk.

**Figure 7:**
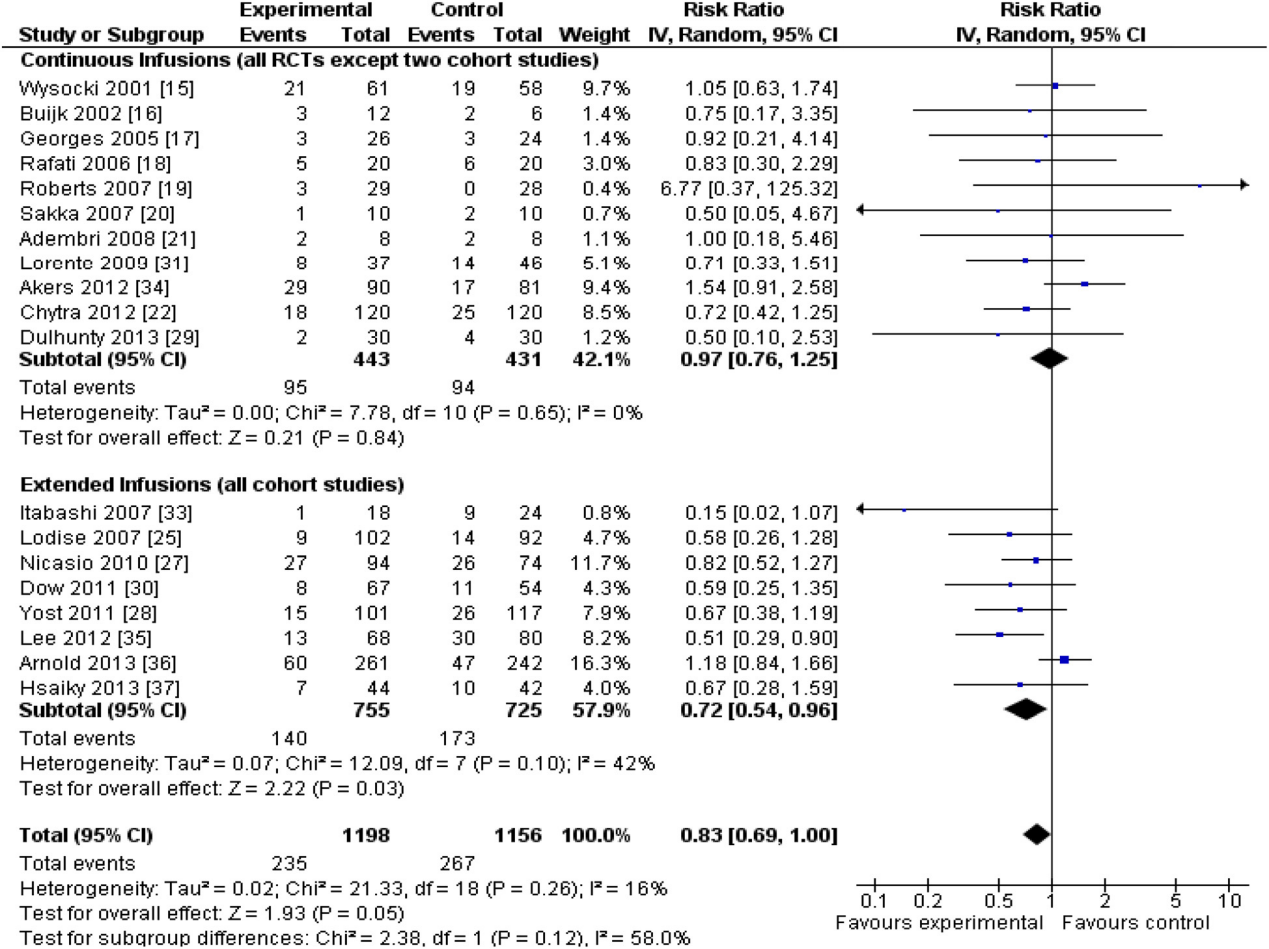
**Effects of pharmacodynamic-based antibiotic dosing on mortality comparing continuous with extended-infusion subgroups.** The continuous-infusion studies included nine RCTs [15-22,29] and two cohort studies [31,34], whereas the extended-infusion studies included only cohort studies. Individual-study RRs with 95% CIs are shown as squares with lines, and pooled RRs with 95% CIs, calculated by using random-effects models both overall and separately for each subgroup, are shown as diamonds. The interaction *P* value, calculated by using a *Z* test, testing for subgroup differences between continuous and extended infusion studies, did not achieve statistical significance (*P* = 0.12). Weight refers to the weighting of each individual study to the overall pooled RR. CI, confidence interval; IV, inverse variance; RCT, randomized controlled trial; RR, relative risk.

Examining effects by types of antibiotics (Figure 6), only studies involving piperacillin/tazobactam (or piperacillin alone) clearly demonstrated a survival advantage for the intervention group (five studies [18,25,28,31,35], *n* = 683; RR, 0.62; 95% CI, 0.46 to 0.85; *P* = 0.003; *I*^*2*^ = 0%), although only one of five studies in this subgroup was an RCT [18]. Studies involving carbapenems almost demonstrated a survival advantage for the intervention group (four trials [20,22,33,37]; *n* = 388; RR, 0.64; 95% CI, 0.41 to 1.00; *P* = 0.051; *I*^*2*^ = 0%), with two of four studies being RCTs [20,22]. With respect to type of intervention, extended infusions, all of which were cohort studies, improved survival (eight studies [25,27,28,30,33,35-37]; *n* = 1,580; RR, 0.72; 95% CI, 0.54 to 0.96; *P* = 0.03; *I*^*2*^ = 42%). Improved survival in the studies using continuous infusions did not achieve statistical significance (nine RCTs [15-22,29] and two cohort studies [31,34], *n* = 874; RR, 0.97; 95% CI, 0.76 to 1.25; *P* = 0.84; *I*^*2*^ = 0) (Figure 7).

## Discussion

Pooled results from small RCTs suggest that PDD, by using primarily continuous or extended infusions of antibiotics, reduces clinical failure rates and ICU LOS in critically ill patients when compared with traditional dosing methods. Reduced mortality rates almost achieved statistical significance when the results of RCTs were combined with cohort studies.

Unlike previous meta-analyses, our systematic review included only data from critically ill patients, stratified results by RCTs versus cohort studies, included all clinically used antibacterial agents, and a larger number of studies. We were able to demonstrate a statistically significant improvement in clinical outcomes (reduced clinical failure rates) and ICU LOS, even when exclusively methodologically more-rigorous RCT data are pooled. Three previous meta-analyses, each with fewer studies, included both critically ill and non-critically ill patients and found somewhat different results. Two of these meta-analyses found either no benefit [5,6] or that clinical outcomes were improved only when the same dose of antibiotic was given as continuous infusions when compared with intermittent infusions [6]. Our more comprehensive and updated search included all of the RCTs in ICU found in previous systematic reviews plus additional studies, which may have contributed to these differences.

Similar to the most recent meta-analysis [7], we also found that mortality improvement was seen with continuous/extended infusions of only piperacillin/tazobactam and carbapenems in ICU patients, albeit largely because of data from non-RCTs.

Our pooled results, at least among RCTs, were consistent between studies. This lack of statistical heterogeneity occurred despite significant differences between studies in types of antibiotics used, interventions studied (that is, extended or continuous infusions, or other pharmacodynamic-based dosing strategies), dosages of antibiotic used (that is, whether both arms of the study received the same dose of antibiotic, whether loading doses were given), types of organisms or infections studied, and whether concomitant pharmacokinetic data (that is, therapeutic drug monitoring) was also performed to validate the dosing strategies. We found piperacillin/tazobactam to be the most studied antibiotic, and the only one that resulted in a clear improvement in mortality, albeit largely because of cohort studies. In our study, extended infusions but not continuous infusions demonstrated a statistically significant reduction in mortality. This is inconsistent with the theoretical background, given that extended infusions may not result in serum antibiotic concentrations that are above the minimum inhibitory concentration (MIC) of the infecting pathogen throughout the entire dosing interval, and our findings may be due to methodologic differences, given that all of the extended-infusion studies were nonrandomized, whereas all but two of the continuous-infusion studies were RCTs. However, although for antibiotics such as beta-lactams and carbapenems, the commonly accepted PD parameter associated with improved cure rates are free drug concentration above MIC for 40% to 70% of the dosing interval, these parameters have not been subjected to rigorous clinical evaluation in multiple studies, and their validity was recently challenged [90].

In addition, it is well known that pharmacokinetic parameters are highly variable in critically ill patients because of a variety of factors [91], and thus whether any PD targets were actually attained by any interventions should ideally be confirmed by using actual pharmacokinetic measurements in each individual study, to better correlate with clinical and other end points. For example, augmented renal clearance, seen in some critically ill sepsis and trauma patients [92], might lead to an inability to achieve concentrations above the MIC because of greater clearance in some patients, and this would have a greater impact on continuous versus extended infusions.

As evident from the list of studies included in this meta-analysis, PDD strategies are not a new concept. Indeed, the concept of dual-individualization incorporating both patient PCK and bacterial PD information to arrive at dosage regimen dates back to the 1980s [23]. Even the concept of extended or continuous infusions would benefit from individualization by using patient-specific PCK parameters and organism-specific MIC to verify that these infusions did indeed reach the PD target. Given the intense resources required for such an intervention (that is, infrequently reported PCK of antibiotics in ICU patients, or bacteria-specific MIC for each infection), this concept has not been universally adopted. More recently, given the increase in bacterial resistance and dearth of new antibiotics, significant attention has been paid to optimizing use of currently existing antibiotics through, for example, extended/continuous infusions. Practically speaking, it is still not an accepted standard of practice for all institutions to report MICs for all organisms despite having these MICs determined by automated systems because of errors associated with automated techniques, and there are still a large number of unknowns when it comes to PCK parameters in ICU patients. Therefore to translate the knowledge truly from the plethora of *in vitro* / Monte Carlo-type studies to actual ICU patients, significant system changes and further research, as previously outlined, must occur. This systematic review of primarily small, single-center studies of critically ill patients, a patient population that is most likely to benefit because of their severity of illness and increased potential for infections with more-resistant organisms, suggests that PDD may lead to improved patient-centered clinical outcomes and supports the conduct of more adequately powered and rigorously performed RCTs to confirm these findings.

The strengths of our study include the use of rigorous systematic review and meta-analytic methods consistent with PRISMA guidelines [93], including a reproducible and comprehensive literature-search strategy without language restrictions, clearly defined inclusion criteria, duplicate citation review, data abstraction, and quality assessment of individual studies, and a predefined statistical-analysis plan. Our meta-analysis also included more studies of critically ill patients: previous meta-analyses included only five to seven studies enrolling primarily critically ill patients, of which only two to six were RCTs [5-7], whereas our meta-analysis included 26 studies enrolling primarily critically ill patients, of which 13 were RCTs.

Our study also has limitations. The numbers of patients enrolled in the selected studies were relatively small, and most of the RCTs were unblinded and single center, with only a minority reporting on quality indicators, such as allocation concealment, intention-to-treat analysis, and losses to follow-up after randomization. This makes further subgroup analysis not useful, given the small sample size in each study and the types of studies. To be comprehensive, we included all antibacterials, all study types, and all dosages of antibiotics and also studies targeting different PD end points, which resulted in clinical heterogeneity among included studies. Surprisingly, the pooled results, at least among RCTs, demonstrated no statistical heterogeneity; however, tests for heterogeneity have lower statistical power when the number of trials is small. Clinical cure is a subjective outcome that was defined by each study’s authors, and potentially subject to bias, given that the studies were mainly unblinded [94], and the microbiologic causes of infections were different, and appropriateness of empiric antibiotics, a key determinant of outcomes, was not reported. Even a moderately sized additional RCT could negate the statistically significant improvement in this outcome. For example, a recently completed blinded placebo-controlled RCT in critically ill patients with ventilator-associated pneumonia [95], which did not meet our inclusion criteria because it compared two different antibiotics for different durations of therapy (extended (4-hour) dose doripenem for 7 days versus intermittent dose imipenem/cilastatin for 10 days), found higher clinical failure rates in the extended-dose doripenem group (43/79 (54%) versus 38/88 (43%)). Adding data from this trial to our pooled result would make the improved clinical failure rates among the continuous/extended RCTs no longer statistically significant: eight RCTs, *n* = 732; RR, 0.81; 95% CI, 0.57 to 1.15; *P* = 0.24. It would also eliminate statistically significant mortality improvements in the subgroup of extended-infusion cohort studies, and the subgroup of carbapenem studies.

In addition, almost all studies included in this review permitted the use of concomitant antibiotics [12,14-19,21,22,24-31,34-37], whereas the remainder did not specifically report on whether their use was permitted [13,20,23,32,33]. This use of concomitant antibiotics may have contributed to reduced differences in outcomes between groups. We also did not conduct our analysis controlling for differences in antibacterial dosing regimens (for example, with or without loading doses) or patient severity of illness. The latter would require patient-level data that would be challenging to acquire.

## Conclusions

In conclusion, pooled results from small RCTs suggest that PDD reduces clinical failure rates and ICU LOS in critically ill patients, and may reduce mortality rates when the results of RCTs are combined with cohort studies. Given the limitations of our review, these findings support the conduct of future adequately powered and well-designed RCTs to confirm these findings for this important clinical question.

## Key messages

• Pooled analysis of randomized controlled trials suggests that continuous/extended infusions of antibiotics in critically ill patients improve cure rates, length of stay, and possibly mortality.

• This study adds to the current body of literature by focusing on critically ill patients and including a larger number of studies without restriction on type of antibiotics.

## Abbreviations

CI: Confidence interval; ICU: Intensive care unit; LOS: Length-of-stay; MIC: Minimum inhibitory concentration; PCK: Pharmacokinetic; PD: Pharmacodynamic; PDD: Pharmacodynamic-based dosing; RCT: Randomized controlled trial; RR: Relative risk.

## Competing interests

On behalf of all authors, the corresponding author states that there is no competing interest.

## Authors’ contributions

CC contributed to the design of the study, data collection, data analysis, and wrote the initial draft of the manuscript and revised subsequent drafts. AL contributed to data collection and analysis and contributed to the draft of manuscripts. JF contributed to the design of the study, data collection and analysis, and also to the revisions of the manuscripts. All authors approved the final draft of the manuscript.

## Supplementary Material

Additional file 1 **Search Strategy.** Description: Detailed search strategy used to identify relevant citations in the MEDLINE database. Similar search strategies were used for the other databases. Click here for file

Additional file 2 **Funnel plot.** Description: Funnel plot comparing the effect measure, relative risk (RR), for the primary outcome of mortality for each study, including both randomized controlled trials and cohort studies, with its precision, expressed as the standard error of the natural logarithm of RR, SE(log(RR)). Click here for file
