# Supplementary material for: Optimal dosing of antibiotics in critically ill patients by using continuous/extended infusions: a systematic review and meta-analysis
Source: Crit Care. 2013 Nov 29;17(6):R279. doi: 10.1186/cc13134 (PMC4056781; doi:10.1186/cc13134)
Supplement: Additional file 1 — Search Strategy. Description: Detailed search strategy used to identify relevant citations in the MEDLINE database. Similar search strategies were used for the other databases. [file cc13134-S1.PDF]

Additional File 1. Detailed Search Strategy*

* Similar search strategy was used for EMBASE, HealthStar, and CINHAL databases

Database: Ovid MEDLINE(R) In-Process & Other Non-Indexed Citations and Ovid MEDLINE(R) <1946 to September 24, 2013>

Search Strategy:

----

1 exp critical illness/ (16702)

2 exp critical care/ (43531)

3 *intensive care/ (8424)

4 exp Intensive Care Units/ (55494)

5 (critical adj2 care).mp. [mp=title, abstract, original title, name of substance word, subject heading word, keyword heading word, protocol supplementary concept, rare disease supplementary concept, unique identifier] (34514)

6 (intensive adj2 care).mp. [mp=title, abstract, original title, name of substance word, subject heading word, keyword heading word, protocol supplementary concept, rare disease supplementary concept, unique identifier] (114908)

7 ICU.mp. (28782)

8 exp Ventilators, Mechanical/ (8232)

9 2 or 3 or 4 or 5 or 6 or 7 or 8 (155657)

10 *Anti-Bacterial Agents/ (148247)

11 anti-bacterial agents/ or exp alamethicin/ or exp amdinocillin/ or exp amdinocillin pivoxil/ or exp amikacin/ or exp amoxicillin/ or exp amoxicillin-potassium clavulanate combination/ or exp amphotericin b/ or exp ampicillin/ or exp anisomycin/ or exp antimycin a/ or exp aurodox/ or exp azithromycin/ or exp azlocillin/ or exp aztreonam/ (275811)

12 anti-bacterial agents/ or exp bacitracin/ or exp bacteriocins/ or exp bambermycins/ or exp bongkrekic acid/ or exp brefeldin a/ or exp butirosin sulfate/ (248763)

13 anti-bacterial agents/ or exp calcimycin/ or exp candicidin/ or exp capreomycin/ or exp carbenicillin/ or exp carfecillin/ or exp cefaclor/ or exp cefadroxil/ or exp cefamandole/ or exp cefatrizine/ or exp cefazolin/ (256846)

14 *anti-bacterial agents/ or exp cefixime/ or exp cefmenoxime/ or exp cefmetazole/ or exp cefonicid/ or exp cefoperazone/ or exp cefotaxime/ or exp cefotetan/ or exp cefotiam/ or exp cefoxitin/ or exp cefsulodin/ (160153)

15 anti-bacterial agents/ or exp cefuroxime/ or exp cephacetrile/ or exp cephalexin/ or exp cephaloglycin/ or exp cephaloridine/ or exp cephalosporins/ or exp cephalothin/ or exp cephamycins/ or exp cephapirin/ or exp cephradine/ (264278)

16 *anti-bacterial agents/ or exp chloramphenicol/ or exp chlortetracycline/ or exp citrinin/ or exp clarithromycin/ or exp clavulanic acid/ or exp clavulanic acids/ or exp clindamycin/ or exp cloxacillin/ or exp colistin/ or exp cyclacillin/ (178866)

17 anti-bacterial agents/ or exp dactinomycin/ or exp daptomycin/ or exp demeclocycline/ or exp dibekacin/ or exp dicloxacillin/ or exp dihydrostreptomycin sulfate/ or exp diketopiperazines/ or exp distamycins/ or exp doxycycline/ or exp echinomycin/ or exp edeine/ or exp enviomycin/ or exp erythromycin/ or exp erythromycin estolate/ or exp erythromycin ethylsuccinate/ (276894)

18 anti-bacterial agents/ or exp filipin/ or exp floxacillin/ or exp fluoroquinolones/ or exp fosfomycin/ or exp framycetin/ or exp fusidic acid/ or exp gentamicins/ or exp gramicidin/ or exp hygromycin b/ or exp imipenem/ (274768)

19 *anti-bacterial agents/ or exp josamycin/ or exp kanamycin/ or exp kitasamycin/ or exp lactams/ or exp lasalocid/ or exp leucomycins/ or exp lincomycin/ or exp lincosamides/ or exp lucensomycin/ or exp lymecycline/ (249950)

20 anti-bacterial agents/ or exp methacycline/ or exp methicillin/ or exp mezlocillin/ or exp mikamycin/ or exp minocycline/ or exp miocamycin/ or exp moxalactam/ or exp mupirocin/ or exp mycobacillin/ (246207)

21 *anti-bacterial agents/ or exp nafcillin/ or exp natamycin/ or exp nebramycin/ or exp neomycin/ or exp netilmicin/ or exp netropsin/ or exp nigericin/ or exp nisin/ or exp norfloxacin/ or exp novobiocin/ or exp nystatin/ (166313)

22 *anti-bacterial agents/ or exp ofloxacin/ or exp oleandomycin/ or exp oligomycins/ or exp oxacillin/ or exp oxytetracycline/ or exp paromomycin/ or exp penicillanic acid/ or exp penicillic acid/ or exp penicillin g/ or exp penicillin g benzathine/ or exp penicillin g procaine/ or exp penicillin v/ or exp piperacillin/ or exp pivampicillin/ or exp polymyxin b/ or exp polymyxins/ or exp pristinamycin/ or exp prodigiosin/ (196917)

23 exp rifabutin/ or exp rifamycins/ or exp ristocetin/ or exp rolitetracycline/ or exp roxarsone/ or exp roxithromycin/ or exp rutamycin/ (20391)

24 exp sirolimus/ or exp sisomicin/ or exp spectinomycin/ or exp spiramycin/ or exp streptogramin a/ or exp streptogramin group a/ or exp streptogramin group b/ or exp streptogramins/ or exp streptomycin/ or exp streptovaricin/ or exp sulbactam/ or exp sulbenicillin/ or exp sulfamethoxypyridazine/ (39596)

25 exp talampicillin/ or exp teicoplanin/ or exp tetracycline/ or exp thiamphenicol/ or exp thienamycins/ or exp thiostrepton/ or exp ticarcillin/ or exp tobramycin/ or exp troleandomycin/ or exp tunicamycin/ or exp tylosin/ or exp tyrocidine/ or exp tyrothricin/ or exp valinomycin/ or exp vancomycin/ or exp vernamycin b/ or exp viomycin/ or exp virginiamycin/ or exp beta-lactams/ (146797)

26 antibiotic.mp. (145401)

27 alamethicin$/ or amdinocillin$/ or amdinocillin$/ or amikacin$/ or amoxicillin$/ or amphotericin$/ or ampicillin$/ or anisomycin$/ or antimycin$/ or aurodox/ or azithromycin$/ or azlocillin$/ or aztreonam$/ (44126)

28 bacitracin$/ or bambermycins$/ or bongkrekic$/ or brefeldin$/ or butirosin$.mp. [mp=title, abstract, original title, name of substance word, subject heading word, keyword heading word, protocol supplementary concept, rare disease supplementary concept, unique identifier] (5318)

29 calcimycin$/ or candicidin$/ or capreomycin$/ or carbenicillin$/ or carfecillin$/ or cefaclor$/ or cefadroxil$/ or cefamandole$/ or cefatrizine$/ or cefazolin$/ (18591)

30 cefuroxime$/ or cephacetrile$.mp. or cephalexin$/ or cephaloglycin$/ or cephaloridine$/ or cephalosporin$/ or cephalothin$/ or cephamycin$/ or cephapirin$/ or cephradine$.mp. [mp=title, abstract, original title, name of substance word, subject heading word, keyword heading word, protocol supplementary concept, rare disease supplementary concept, unique identifier] (8118)

31 chloramphenicol$/ or chlortetracycline$/ or citrinin$/ or clarithromycin$/ or clavulanic acid$/ or clindamycin$/ or cloxacillin$/ or colistin$/ or cyclacillin$.mp. [mp=title, abstract, original title, name of substance word, subject heading word, keyword heading word, protocol supplementary concept, rare disease supplementary concept, unique identifier] (37300)

32 dactinomycin$/ or daptomycin$/ or demeclocycline$/ or dibekacin$/ or dicloxacillin$/ or dihydrostreptomycin$/ or exp diketopiperazine$/ or distamycin$/ or doxycycline$/ or echinomycin$/ or edeine$/ or enviomycin$/ or erythromycin$.mp. [mp=title, abstract, original title, name of substance word, subject heading word, keyword heading word, protocol supplementary concept, rare disease supplementary concept, unique identifier] (54828)

33 filipin$/ or floxacillin$/ or fluoroquinolone$/ or fosfomycin$/ or framycetin$/ or fusidic acid$/ or gentamicin$/ or gramicidin$/ or hygromycin b$/ or imipenem$.mp. [mp=title, abstract, original title, name of substance word, subject

heading word, keyword heading word, protocol supplementary concept, rare disease supplementary concept, unique identifier] (43334)

34 methacycline$/ or methicillin$/ or mezlocillin$/ or mikamycin$/ or minocycline$/ or miocamycin$/ or moxalactam$/ or mupirocin$/ or mycobacillin$/ (11201)

35 nafcillin$/ or natamycin$/ or nebramycin$/ or neomycin$/ or netilmicin$/ or netropsin$/ or nigericin/ or nisin$/ or norfloxacin$/ or novobiocin$/ or nystatin$.mp. [mp=title, abstract, original title, name of substance word, subject heading word, keyword heading word, protocol supplementary concept, rare disease supplementary concept, unique identifier] (20493)

36 ofloxacin$/ or oleandomycin$/ or oligomycin$/ or oxacillin$/ or oxytetracycline$/ or paromomycin$/ or penicillin$.mp. or piperacillin$/ or pivampicillin$/ or polymyxin$/ or pristinamycin$/ or prodigiosin$/ [mp=title, abstract, original title, name of substance word, subject heading word, keyword heading word, protocol supplementary concept, rare disease supplementary concept, unique identifier] (93176)

37 rifabutin$/ or rifamycin$/ or ristocetin$/ or rolitetracycline$/ or roxarsone$/ or roxithromycin$/ or rutamycin$.mp. [mp=title, abstract, original title, name of substance word, subject heading word, keyword heading word, protocol supplementary concept, rare disease supplementary concept, unique identifier] (3591)

38 talampicillin$/ or teicoplanin$/ or tetracycline$/ or thiamphenicol$/ or thienamycin$/ or thiostrepton/ or ticarcillin$/ or tobramycin$/ or troleandomycin$/ or tunicamycin$/ or tylosin$/ or tyrocidine$/ or tyrothricin$/ or valinomycin$/ or vancomycin$/ or vernamycin$/ or viomycin$/ or virginiamycin$/ or beta-lactams$/ or doripenem$.mp. or meropenem$.mp. or meropenem$.mp. or exp ciprofloxacin/ or ciprofloxacin$.mp. or exp ofloxacin/ or ofloxacin$.mp. or levofloxacin$.mp. or moxifloxacin$.mp. or gatifloxacin$.mp. [mp=title, abstract, original title, name of substance word, subject heading word, keyword heading word, protocol supplementary concept, rare disease supplementary concept, unique identifier] (81865)

39 10 or 11 or 12 or 13 or 14 or 15 or 16 or 17 or 18 or 19 or 20 or 21 or 22 or 23 or 24 or 25 or 26 or 27 or 28 or 29 or 30 or 31 or 32 or 33 or 34 or 35 or 36 or 37 or 38 (590981)

40 (glycopeptide$ or fluroquinolone$ or cabapenems$ or co-azoldinone$).mp. or llinezolid$/ or lipopeptide$.mp. or Glycycline$.mp. or tigecycline$.mp. or lincosamide$.mp. or malrolide$.mp. or streptogramin$.mp. [mp=title, abstract, original title, name of substance word, subject heading word, keyword heading word, protocol supplementary concept, rare disease supplementary concept, unique identifier] (20012)

41 39 or 40 (602953)

42 pharmacodynamic$.mp. (30786)

43 exp pharmacokinetics/ and ((pharmacodynamic$ or dual diagnosis).mp. or *time factors/) [mp=title, abstract, original title, name of substance word, subject heading word, keyword heading word, protocol supplementary concept, rare disease supplementary concept, unique identifier] (5553)

44 exp antibacterial agents/ad and exp time factors/ (7847)

45 exp drug administration schedule/ and (pk.fs. or exp antibacterial agents/) (15452)

46 or/42-45 (52059)

47 1 or 9 (162470)

48 41 and 47 (10764)

49 41 and 46 and 47 (656)

50 monobactam:.tw. (637)

51 monobactam.mp. (353)

52 carbapenem:.mp,tw. (6992)

53 beta lactam.mp. or exp beta-Lactams/ (113379)

54 50 or 51 or 52 or 53 (115628)

55 41 or 54 (604762)

56 ((continuous$ or discontinuous$ or intermittant or extended or bolus) adj3 (administrat$ or dosing$ or dosage$ or dosing$)).mp. [mp=title, abstract, original title, name of substance word, subject heading word, keyword heading word, protocol supplementary concept, rare disease supplementary concept, unique identifier] (9606)

57 47 and 55 and 56 (87)

58 57 not 49 (39)

59 exp Infusions, Parenteral/ (84116)

60 47 and 55 and 59 (372)

61 60 not 57 (341)

62 limit 61 to animals (9)

63 limit 61 to case reports (38)

64 61 not (62 or 63) (295)

65 remove duplicates from 64 (277)
